# An Account of the Epidemic Catarrhal Fever, or Influenza, in Manchester, Together with Some General Remarks on This and Similar Epidemics

**Published:** 1803-06-01

**Authors:** Samuel Argent Bardsley

**Affiliations:** Physician to the Manchester Infirmary, Dispensary, and Lunatic Hospital


### *An Account of the* EPIDEMIC CATARRHAL FEVER, *or* INFLUENZA, *in Manchester, together with some general Remarks on this and* SIMILAR EPIDEMICS; *communicated by* SAMUEL ARGENT BARDSLEY, M. D. *Physician to the Manchester Infirmary, Dispensary, and Lunatic Hospital.*

I Make no doubt but the prevalence of the present epidemic disorder will attract the attentive observation of a great many of your medical Correspondents. It is only by collecting and comparing facts and observations, from practitioners in various parts of the kingdom, that a correct and complete history of this widely-extended disease can be expected to be established. To assist in accomplishing part of this plan, I propose, first, to describe such particulars relative to its history and treatment as have been furnished by my own observations and experience, and then to subjoin a few remarks on the present and other similar epidemic disorders. The

The Influenza appeared in Manchester as early as the first week in February, but extended itself for some weeks in only a partial and confined degree; I met, however with no instance of the disease, either in public or private practice, prior to the beginning of March; many other practitioners likewise assign this latter date as the period of its commencement: yet I am able to state, upon the unquestionable authority of my friend, Dr. Percival, that the disease manifested itself, in his own family, on the 4th of February; and from the best of his recollection, it occurred in several cases during the early part of that month. It does not, however, appear to have spread throughout all parts of the town, until the second week of March.

I find it exerted its greatest influence among the lower order of the inhabitants, between the 26th of March and the 2d of April. This is evident from the number of patients, entered into the home-patient books of the Infirmary, having exceeded in a four-fold proportion that of any other week since the commencement of the year.—Most of these cases which came under my care, were of the worst kind, and such as required admission into the Fever Wards, or House of Recovery. A still greater number of the poor, who suffered by the disease in its milder form, were treated as out-patients of the Dispensary. But its influence was by no means partial; in two respectable families, all the servants, to the number of seven in one instance and six in the other, were afflicted at the same time. Heads of opulent families and very young children, form a comparative exception to its indiscriminate attack.

The Faculty agree in opinion, (as far as my inquiries have extended) that no other instance of an epidemic, so generally prevalent, has been noticed in this town. It is not an exaggerated calculation, to estimate the number of persons afflicted with this malady at one-tenth of the whole population. One practitioner has attended, within a month, upwards of three hundred patients, chiefly amongst the middle class of the inhabitants. The epidemic, in its genuine form, exhibited the following symptoms: Spontaneous weariness and languor, succeeded by slight shiverings, with alternate flushings of heat, first attack the patient; he then complains of a deep seated pain in the course of the frontal sinuses, accompanied, for the most part, with sneezing and a profuse discharge of lymph from the nose and eyes. In the space of a few hours, acute darting pains in the muscles subservient to respiration, attended with a tickling cough and hoarseness, frequently occur. As the disease advances, the patient complains of much anxiety about the præcordia, dull, aching pains in the back and knee-joints, and of great debility, languor, and depression of spirits. The tongue is early covered with an extremely white mucus, and has the appearance of having been suffused with milk. The pulse is rather small and quick, (seldom if ever hard and full) its number of beats exceeding an hundred, and often during the evening exacerbations one hundred and twenty in a minute. The tongue being moist, little or no complaint is made of thirst. Costiveness, for the most part, prevails. The appetite is not only entirely lost, but a fixed loathing to any solid food is expressed. A degree of cardialgic nausea, often succeeded by vomiting and griping pains in the bowels, torments the patient upon the second or third day of the attack. On the third evening, and sometimes as late as the fourth, the disease seems to have attained its acme. The cough, fever, and debility, with increased irritation about the fauces and breast, greatly harrass the patient; and it is only after a night spent in restless inquietude, with delirious wanderings, that he enjoys from a slight repose in the mornihg, some respite from these distressing symptoms. A cough, generally moist, with impeded respiration, and pricking heat in the throat and breast, continues after the febrile symptoms have, in a great degree, subsided. The appearance of the urine on the fifth or sixth day, denotes the diminution of the fever. It deposits a copious sediment, and changes from rather a pale and limpid hue to a deeper and more natural colour. Great prostration of strength is still perceived, and the patient is seized with sudden faintings upon any attempt to raise him from an horizontal posture.

The above description is only intended to apply to the genuine, unmixed form of the epidemic. Modified by age, sex, and temperament, and (admitting it to be contagious) by the circumstances under which contagion was communicated, it manifested a remarkable diversity in its effects upon different subjects; yet, in every case, some degree of resemblance might be observed. Its distinctive character was never completely lost. A sudden prostration of strength, lassitude, constriction, and pain in the forehead, together with watery discharges from the eyes and nose, feverish chilliness and heat, unaccompanied with a correspondent degree of derangement in the arterial system the existence of some or all of these symptoms, in a combined combined or separate state, sufficiently characterize the epidemic.

A great difference was noticed in the nature and degree of the local affection of the breast and fauces. In some, the mucous membrane was but slightly inflamed; in others, great pain and difficulty in breathing, and universal rawness and soreness in the trachea and chest, indicated more extensive inflammation. But in two* instances only evident symptoms of pneumonic inflammation occurred. Many were solely affected with the prominent, and indeed almost pathognomonic symptoms, of intense pain in the head, and general debility; either sickness or diarrhœa, with transient shiverings and debility, unaccompanied with any catarrhal affection, marked the existence of this disorder in many delicate and irritable constitutions.

The medical treatment of this Epidemic admitted of considerable variety; yet from its prevailing character of debility, as manifested by a weak and quick pulse, disturbed state of the stomach and bowels, and general derangement of the animal functions, the chief attention was directed to moderate the febrile symptoms, and to support the powers of life. Many, it is true, required little or no medical aid; but most of the patients, who fell under my care in the House of Recovery, laboured under the disease in its most aggravated and complicated state. Emetics were found highly beneficial on the first attack; indeed, the frequent occurrence of spontaneous nausea and sickness pointed out their use. They scarcely ever failed to relieve the urgent symptoms of pain in the head and stricture of the breast. To obviate costiveness, and at the same time to cleanse the primæ viæ, moderate doses of calomel with rhubarb and antimonial powders combined, were exhibited with excellent effects. When much pain and soreness in the head and breast were felt, attended with a hot and dry skin, either pediluvia or fomentations of the feet and legs with flannels wrung out of hot water, sensibly relieved the patient, by exciting a gentle moisture

---

* One of the cases proved fatal. The patient, a boy of fifteen, had laboured under cough, dyspnœa, and slight hectic fever, previously to the attack of the epidemic. On opening the thorax, soon after his death, strong adhesions were discovered between the left side of the lungs and the pleura; marks of inflammation extended to the pericardium, and even to the heart, its apex being found covered with coagulable lymph. On cutting into the left lobe of the lungs, a considerable quantity of purulent matter oozed out of its cellular substance.

on the surface of the body. To moderate the febrile symptoms, small doses of antimonial wine, combined with spt. æther. nitros. and neutralized ammonia, were almost invariably employed. The local affection of the breast yielded almost uniformly to the application of blisters; even when the pain in the chest was severe and deep seated, attended with impeded respiration and a teazing cough, this remedy seldom failed in affording speedy relief. If an excruciating pain and confusion of the head, turgidity in the vessels of the eyes, and great impatience of light, indicated a more than usual determination of blood to the brain; bleeding with leeches at the temples, and blistering the forehead and nape of the neck, were successfully put in practice. The patient was ordered to be kept in bed, with his head and shoulders moderately raised, until the giddiness of the head and general debility were removed. Opiates were seldom employed during the first stage of the disorder, as they had a tendency to exasperate the complaints of the head and chest, and increase restlessness and feverish heat. When the cough was incessant, and sharp wandering pains affected the breast and muscles subservient to respiration, attended with a rapid and somewhat hard pulse, the addition, in small doses, of the tincture of digitalis, to the saline and diaphoretic medicines already mentioned, not only mitigated the cough, but likewise promoted expectoration, and induced sleep. The usual degree of tickling cough, and soreness of the fauces and breast, was relieved by the frequent use of barley decoction, with gum arabic; or linseed tea, acidulated and rendered palatable by liquorice root. Oily medicines were neither useful nor much relished by the sick. Wine, in gruel or whey, formed a powerful auxiliary in supporting the strength, and preserving a moderate and equable moisture on the skin. In very few cases did symptoms of local or general excitement prevent its administration; indeed, it was found necessary among the Infirmary patients, to counteract febrile debility, after due evacuations of the stomach and intestines, by a light nutritious diet, and a moderate use of wine. Tonic bitters, with the mineral acids, proved useful in checking profuse discharges by the skin or lungs, and contributed likewise powerfully to restore the general health. The lancet was very seldom employed; in only two instances it was deemed necessary to use general bleeding; and even topical depletion of the vessels was rarely adviseable. To subdue the obstinate cough and copious expectoration pectoration of aged people, and those afflicted with asthma, or other pulmonic complaints, small combined doses of opium, digitalis, and calomel, along with bark and other tonics, were generally employed. By the above method of treatment, varied according to circumstances, the worst cases were discharged cured from the House of Recovery, in about an average period of twelve days. Notwithstanding the universality of the epidemic in this crouded and very populous town, few have fallen victims to its severity, and those who died were for the most part old, asthmatic, or other debilitated subjects. Within the last fortnight I have seen several instances where this disease degenerated into typhus, and the patients incurred great risk of recovery. Under proper management, however, it may justly be termed a disease of safe and speedy termination. Relapses do not appear to have been very frequent. I have met with but few who have suffered a repetition of the worst symptoms of the disorder. It has now (the 26th of April) almost disappeared in this town; but is exerting its influence in full vigor throughout the populous places of the vicinity.

*General Remarks on the present and other similar Epidemics.*

The similarity of the present Epidemic Catarrh to those which, at various periods, have infested Europe and other parts of the globe, cannot be disputed. Dr. Cullen, in his Nosology, has quoted authorities to prove the occurrence of a contagious malady of this kind, at no less than thirty distinct periods, from the year 1323 to 1767. On referring to these authorities, its different degrees of resemblance may be traced between the histories of all these several epidemics. But the years 1762 and 1782,* furnish the strongest proofs of identity between the contagious diseases then prevailing in this island, and that which now exists.—What are the remote and predisposing causes of this widely extended malady? and in what does its nature and essential character consist? are questions concerning which medical writers greatly differ. The present epidemic cannot have originated, in this part of the kingdom, from any remarkable severity of weather, or sudden changes in the temperature of the atmosphere. No season has been apparently less unfriendly to the human constitution than the whole of the late winter and early part of spring. This

* See Medical Transactions, Vol. ii. p. 54.

will appear from the following summary statement of the weather, since the year commenced.

January. Till the 10th, mild and cloudy.

February. From January 10 to February 12, uniform frost, without much intension or remission. Mostly fine and clear with little snow.

After the 12th, showery weather, with gleams, to the end of the month.

March. For two days heavy rain; from this to the middle of the month, frosty nights but mild days, with little snow or rain; the rest of the month rather warm for the season, and little rain.

April. Till the 17th uncommonly fine and warm for the season; remarkably drying winds.

The barometer has been no way uncommon during the season; the winds have been principally N. E. and S. W. as usual.

If alternate severe frosts and chilly moist weather, (the usual sources of common catarrh) were the origin of contagious catarrhal fever, how are we to account for the universal epidemics of 1762 and 1782, prevailing during uncommonly warm and steady weather, in the months of May and June? Moreover, that remarkable epidemic catarrh of 1580, (which according to Sennertus, "Totam pene Europam, imo fere omnes mundi regiones pervagata est,") chiefly raged during the sultry weather of autumn. We must therefore enquire for some other cause than a change in the sensible qualities of the air, to explain the origin of this disease. That it is gradually disseminated over large portions of the globe, from contagious particles in the atmosphere, is an hypothesis generally maintained. The numbers attacked at the same time by the disorder, and the enlarged sphere of its activity, are facts which seem to support this opinion. Yet when we consider the confined operation of the atmosphere in conveying contagious particles, and how soon they are dissipated or diluted, if not chemically changed, by admixture with this fluid, so as to render them totally inert, and incapable of producing disease, (facts strikingly exemplified in the instances of plague and small-pox) we shall hesitate in giving implicit assent to this doctrine. If the general mass of air were contaminated, how are we to account for the slow and gradual progress of the malady? A cause so universal ought to have produced correspondent effects. All the sick should have been afflicted nearly at the same time; yet it appears to have raged in Paris some weeks previous to to its appearance in London; and the same length of time was occupied in its progress from the latter city to Manchester. Its capability of being conveyed from one human subject to another, or contagious nature, has been equally a matter of dispute; but that it is a disease of a contagious kind, arising from a specific materies morbi, and readily communicable from one person to another, is rendered probable from various facts, as well as from its analogy to other contagious disorders. Its appearance here, although rapid, was progressive. It spread like other infectious maladies, more particularly among those exposed by their greater intercourse with each other to the danger of contagion. Female domestics, and the inhabitants of the nursery, seldom escaped its influence.

A gentleman of this town returned from London, in the third week of March, while labouring under Influenza. He found his family all well; and unconscious of the infectious nature of his complaint, he bestowed the usual caresses upon his children. Three of them sickened the next day, and two more on that following.

But the most compleat and satisfactory evidence of the propagation of this disease by human contagion, (I had almost said the only mode in which it is communicated) is derived from what happened in the Manchester Lunatic Hospital; none of the patients were afflicted with the epidemic (although permitted to walk out daily in the airing grounds) until the keeper and matron became affected. In consequence of their attention, while labouring under the Influenza, to some elderly patients, whose situation demanded more than common care, they communicated the infection to five of the persons thus circumstanced, while all the rest, to the amount of eighty and upwards, entirely escaped the complaint. The house-servants, who are solely engaged in domestic employments, and are never permitted to associate with the Lunatics, suffered more or less severely by the disorder. Thus it would appear, that a careful exclusion from infected persons is probably the most certain preventive method of obviating a malady so universally prevalent.*

The

* I have just been made acquainted with the high importance of such precautionary means to at least one class of the sick and feeble, predisposed to this infectious malady. Six puerperal women have fallen victims to the attack of the epidemic. One intelligent midwife lost five patients within ten days. This is so important a fact, and shews the necessity so strongly

The nurses of the House of Recovery fell sick, soon after the reception of the first cases of the malady, notwithstanding the usual preventive rules against infection were strictly followed. At Rochdale, I have been assured, the origin of the disease was distinctly traced to some gentlemen who had brought it from Lancaster, where they had attended the assizes.†

The next important question relates to the nature and essential character of the disease. Much diversity of opinion has prevailed on this point. It is however of the utmost importance to be ascertained. From my own observation of its prevailing symptoms in this place, I am disposed to consider it essentially a contagious febrile disease. The sickness, languor, faintings, pain in the head and loins, with a small quick pulse; all demonstrate the character of this fever as arising from a debilitating cause.

This general state of the disorder ought never to be lost sight of in practice. The mucous membrane may be more or less diseased, and even active local inflammation may occasionally prevail; but the former is not necessarily attended with general excitement, and the latter is only to be considered as an anomalous symptom, dependant on peculiarity of constitution, or some other accidental circumstance. Great mischief will arise from confounding its occasional varieties with the permanent and specific character of the disease. Thus we learn from Sennertus,‡ that in the Epidemic Catarrh of 1580, incalculable injury ensued, from a partial view of the *local* symptoms, as indicating a disease of a purely inflammatory kind. He mentions

---

strongly of strict seclusion from the risk of personal infection in all puerperal cases, that it cannot be too generally promulgated. Have similar fatal instances occurred to practitioners in midwifery in other places? And are puerperal women remarkably predisposed to the attack of this epidemic? are queries highly interesting to the Readers and Correspondents of the Medical and Physical Journal.

† The following extract of a communication from Mr. Tomlinson, of this town (a gentleman of accurate observation and extensive practice) still farther strengthens my opinions concerning both the contagious nature and essential character of the epidemic.

"I am confirmed in opinion that it is a *febrile* disease, attended with extreme debility, and so *highly contagious*, that I have often witnessed it spread through large families of those who are in a middle station of life. I have frequently remarked, that debility succeeding intoxication, to be a strikingly predisposing cause, even in those constitutions, apparently, least liable to be attacked. I have not met with many instanc s of its fatality, except in those labouring under previous disorders."

‡ De Febrib. lib. IV. cap. xviii.

mentions the "Tussis sicca, dolor pectoris et præcipue septi transversi; faucium asperitas, ventriculi languor; tandem gravis anhelitus, &c. as symptoms which induced many practitioners to make free use of the lancet; but with such mortal effects, that in the city of Rome alone, where bleeding was promiscuously employed, more than 2000 persons perished. On the contrary, where this evacuation was altogether avoided, the mortality did not exceed more than one in a thousand. "Experientia (says this judicious writer) enim hoc comprobavit; omnes fere mortuos esse, quibus vena aperiebatur."

In the Epidemic Catarrh of 1733,* (one of the most universally prevalent of any upon record) bleeding was generally found to aggravate the symptoms. It raged in Edinburgh, during a cold and severe spring, when inflammatory complaints commonly abound; yet the practitioners of that place observe, "a few of the sick were at once seized with ugly faintings; when bled they recovered more slowly; but when supported by cordials they were soon well." A lesson of caution not to be neglected in the treatment of the present epidemic. Its special observance by the Faculty here has been attended with the best effects. Indeed, the constitutions of the generality of the inhabitants of this and most other populous and manufacturing towns, will not admit of debilitating remedies, in the same degree, as maybe safely put in practice among more temperate and more robust classes of people. It will not be denied that venæsection may be found useful in combating occasional symptoms of this epidemic malady; yet great care must, at all times, be observed in not too far depressing the system by its injudicious employment. The following admonition of Forestus, in his Scholia on the Catarrhal Epidemic of 1580, should be strictly attended to:—"Oportet igitur diligenter distinguere, quibus venæsectio conveniat, & quibus non, juxta indicationes curandi, vel sanguinis mittendi, quæ vel admittant, vel omnino dissuadeant."

---

* See Edinburgh Medical Essays, Vol. II. Art. 2.

